# Modified CLARITY Achieving Faster and Better Intact Mouse Brain Clearing and Immunostaining

**DOI:** 10.1038/s41598-019-46814-4

**Published:** 2019-07-22

**Authors:** Hao Du, Peihong Hou, Liting Wang, Zhongke Wang, Qiyu Li

**Affiliations:** 1 Department of Anatomy, Third Military Medical University, Chongqing, 400038 China; 2 Biomedical Analysis Center, Third Military Medical University, Chongqing, 400038 China; 3 Department of Neurosurgery, Xinqiao Hospital, Third Military Medical University, Chongqing, 400037 China

**Keywords:** Cellular neuroscience, Molecular neuroscience

## Abstract

CLARITY is a hydrogel embedding clearing method that has the advantages of transparency, different tissue compatibility and immunostaining compatibility. However, there are also some limitations to CLARITY as it requires a long time to achieve transparency, and the electrophoresis clearing is complex. Therefore, we aimed to simplify the electrophoresis system and shorten the processing time of CLARITY. In our study, we developed a non-circulation electrophoresis system to achieve easier manipulation of electrophoresis clearing. We modified the original CLARITY protocol in hydrogel embedding methods, clearing buffer and immunostaining. When comparing brains processed by our modified method or the original protocol, we found our modifications permit faster and more efficient clearing and labeling. Moreover, we developed a new clearing method named Passive pRe-Electrophroresis CLARITY (PRE-CLARITY) and a new immunostaining method named Centrifugation-Expansion staining (CEx staining). PRE-CLARITY achieved faster clearing and higher transparency, and CEx staining accomplished intact mouse brain labeling faster. With our modifications to CLARITY, we accomplished intact mouse brain clearing and immunostaining within one week, while this requires weeks to months with the original CLARITY. Our studies would allow high-content tracing and analysis of intact brain or other large-scale samples in a short time.

## Introduction

For centuries, analysis of large-scale tissues mostly depended on microtomes to cut thin sections and provide cellular information at a two-dimensional level. However, biological specimens are intrinsically three-dimensional (3D), so lots of information is lost when cutting them into sections. Therefore, interrogating biological samples at the 3D level is necessary. Recently, there are many innovations to clearing tissue that permit imaging without cutting to acquire 3D information, such as Scale, 3DISCO, SeeDB, iDISCO, CUBIC, CLARITY etc^1^. Combined with various labeling methods, tissue clearing methodologies permit the interrogation of molecular, cellular and system biology across different scales.

CLARITY was reported by *Chung et al*.^2^ in 2013. It transforms tissue into a hydrogel-tissue hybridization and preserves proteins and nucleic acids at their position but eliminates lipids with sodium dodecyl sulfate (SDS) solution to achieve tissue optical transparency^3,4^. Compared with other tissue clearing techniques, CLARITY and its variants have high tissue compatibility and have been successfully used in most organs of the mouse, the whole body of mouse and post-mortem human brain tissue^5–8^. Further, CLARITY-processed tissues permit several rounds of labeling as proteins and nucleic acids are bound to hydrogel network by chemical bonds while other tissue clearing techniques cannot^2^.

However, original CLARITY still has several technical limitations. First, the electrophoresis system is complex so it is difficult to build and maintain. In the clearing system, many devices are needed (sample chamber, chemical resistant tube, refrigerated circulator, filter and peristaltic pump) and the system must be leak-proof. And it builds a closed circulation system, but the circulation does not always work stably as it is easily interrupted by bubbles which form continually during electrophoresis. Second, the original CLARITY is time-consuming as it requires several weeks to achieve clearing and labeling of large-scale specimens. These two main problems impede its application in wider fields. During the years after innovating, many researchers aimed to optimize the CLARITY technique so many variants based on CLARITY emerged^9–14^. In general, many variants focused on two aspects: (1) simplifying the procedures and modifying the system to suit different specimens; and (2) shortening the processing time. As for lipid extraction, the key procedure of tissue clearing, CLARITY and its variants are based on two methods: electrophoresis and passive thermal diffusion. Electrophoretic lipid extraction is faster than passive thermal diffusion, but it requires building a circulation system while passive diffusion does not, which makes it difficult to manipulate. The principle of the staining process is similar to lipid extraction, so it shares problems. Therefore, it is important to find an optimized way that can both shorten the processing time and simplify the system for easy manipulation.

Here, we also focused on the two aspects mentioned above to modify the CLARITY technique. We designed a non-circulation electrophoresis system (NCES) to perform electrophoretic lipid extraction. This system is much simpler to build than that mentioned in the original protocol, and it permits multiple sample clearing at the same time. In addition, we made modifications to the hydrogel embedding methods, clearing buffer and immunostaining method. We reported a new clearing technique named Passive pRe-Electrophroresis CLARITY (PRE-CLARITY) and a new immunostaining method named Centrifugation-Expansion staining (CEx staining). With our modifications for CLARITY, we can achieve an intact mouse brain clearing and immunostaining within one week, while original CLARITY needs at least three weeks for intact mouse brain clearing and labeling^4^. Our study not only simplifies electrophoretic tissue clearing but also saves much time so that the method can be applied in more studies.

## Results

### NCES makes electrophoresis simpler

**Figure 1:**
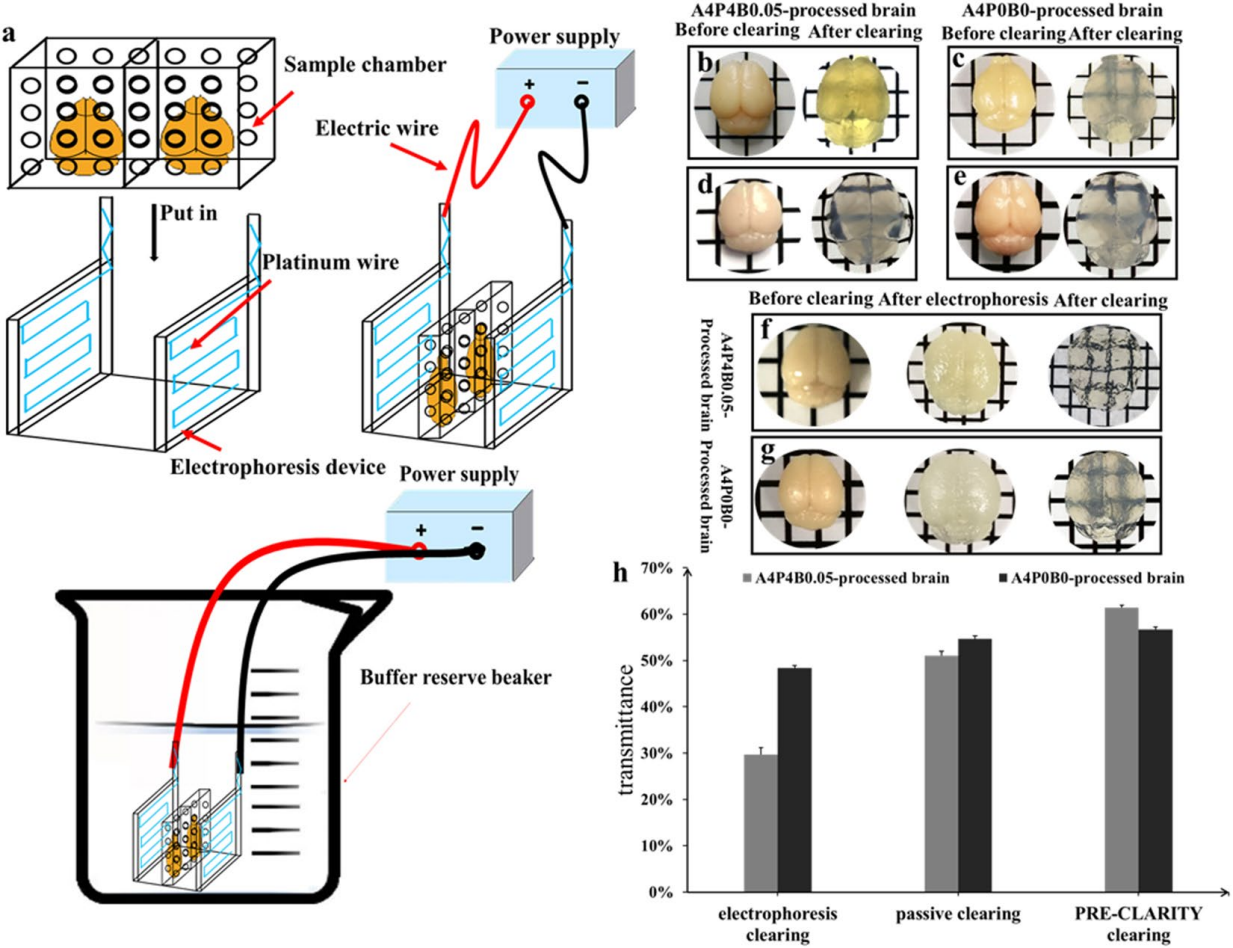
NCES and our modifications to CLARITY provide a simple and efficient method for intact brain clearing. (**a**) Diagram of NCES. The real object and detailed dimensions are provided in Supplementary Fig. S1. (**b**) A4P4B0.05-processed brain before (left) and after (right) 5 days electrophoresis clearing at 25 V/37 °C, without adding 1% α-thioglycerol in clearing buffer. (**c**) A4P0B0-processed brain before (left) and after 60 hours (right) electrophoresis clearing at 25 V/37 °C, with adding 1% α-thioglycerol in clearing buffer. (**d**) A4P4B0.05-processed brain before (left) and after (right) 1 month passive clearing at 50 °C, without adding 5% α-thioglycerol in clearing buffer. (**e**) A4P0B0-processed brain before (left) and after (right) 14 days passive clearing at 37 °C, with adding 5% α-thioglycerol in clearing buffer. (**f**) A4P4B0.05-processed brain before electrophoresis (left), after 1 day electrophoresis at 25 V/37 °C (middle) and after 15 days passive clearing at 50 °C (right). (**g**) A4P0B0-processed brain before electrophoresis (left), after 1 day electrophoresis at 25 V/37 °C (middle) and after 5 days passive clearing at 37 °C (right). (h) Transmittance comparison of A4P4B0.05-processed brain and A4P0B0-processed brain in electrophoresis, passive and PRE-CLARITY clearing (n = 3; Error bars denote standard deviation).

As we mentioned above, the original CLARITY clearing system has several limitations. To simplify the electrophoresis system, we removed the circulation, stored the SDS clearing buffer in a beaker and performed electrophoresis in the beaker (Fig. 1a; Supplementary Fig. S1). Furthermore, we designed a mobile electrophoresis device that allowed observing samples easily during electrophoresis by lifting it. It is also easy to clean byproducts that adhere to electrodes. The electrophoresis device and sample chamber were made by a common PMMA processing plant, and it is less expensive (less than 10 dollars) than using lots of devices (including electrophoresis chamber, peristaltic pump, and filter) to set a complex circulation system (hundreds of dollars). Moreover, the electrophoresis device and sample chamber could be designed in different sizes and structures to suit different specimens and achieve multiple sample clearing at the same time (we tested clearing two brains at the same time). Starting clearing of several samples simultaneously by modifying the electrophoresis device and sample chamber could greatly improve clearing efficiency. Additionally, as the SDS clearing buffer was stored in the beaker, bubbles forming during electrophoresis would float to the water surface and dissolve gradually. In this way, the electrophoresis current would not be interrupted by bubbles. As for byproducts forming during electrophoresis, we noted that they always deposited on the mouse surfaces next to the electrodes. So we hypothesized that most of the byproducts were charged molecules. We set qualitative filter paper in the two sides next to the electrodes, and this prevented byproduct deposition. Before starting electrophoresis, the clearing buffer reserve beaker was put into a constant temperature water bath to maintain the buffer solution at 37 °C.

### Modified CLARITY promotes brain clearing speed and improves transparency

We performed the original CLARITY protocol including electrophoresis (but using NCES rather than the original circulation system) and used passive A4P4B0.05-processed brain clearing as the control group. In our modified methods, we adopted A4P0B0 to perform hydrogel embedding (A4P0B0-processed brain was the experimental group). Moreover, we investigated the effects of PFA post-fixation time and application of α-thioglycerol to tissue clearing. Compared the A4P0B0-processed brain and A4P4B0.05-processed brain, our modifications could promote intact brain clearing speed and enhance transparency both in electrophoresis and passive clearing.

**Figure 2:**
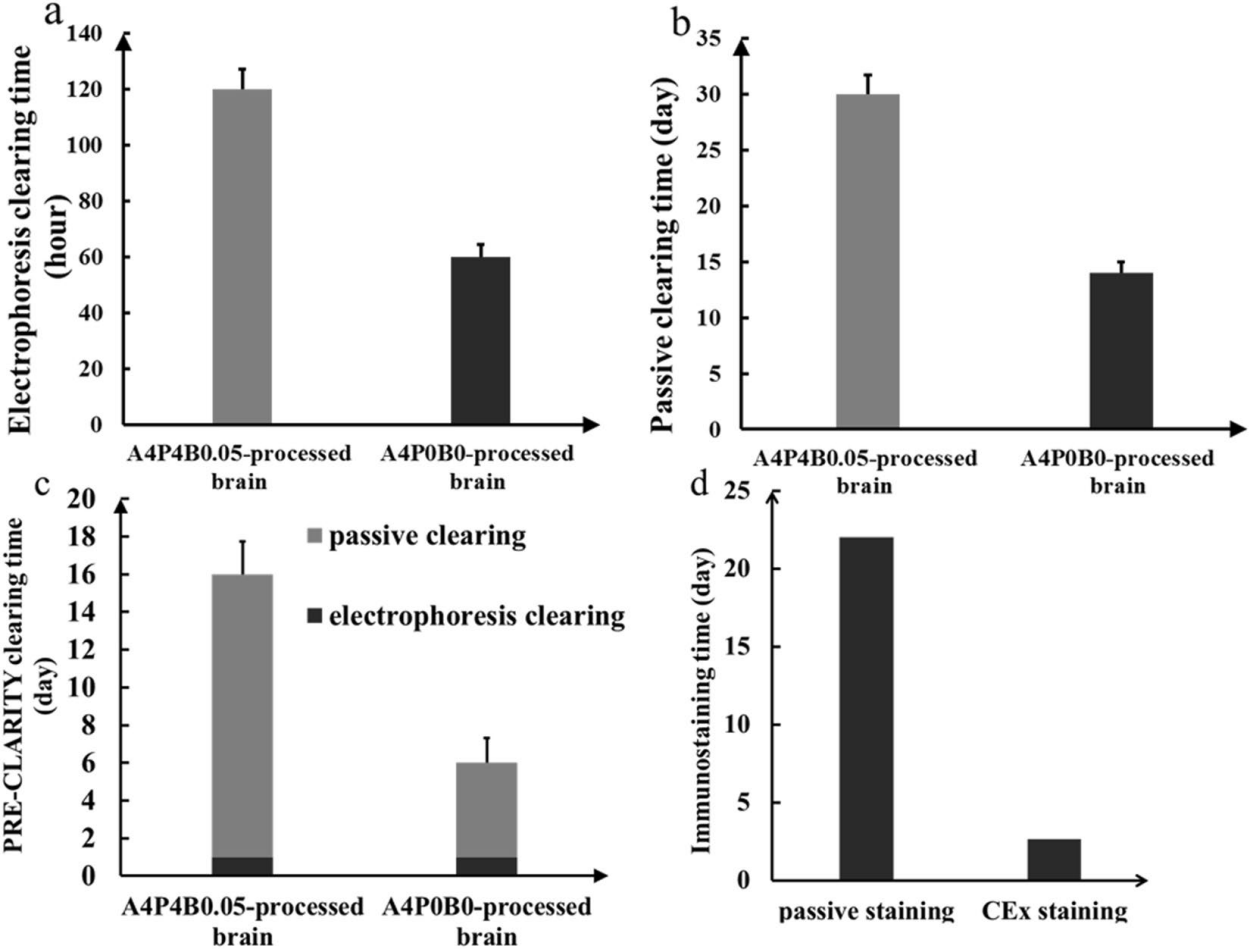
Processing time comparison of original CLARITY and our modified method for brain clearing and immunostaining. (**a**) Clearing time comparison of the A4P4B0.05-processed brain and A4P0B0-processed brain in electrophoresis clearing (n = 3). (**b**) Clearing time comparison of A4P4B0.05-processed brain and A4P0B0-processed brain in passive clearing (n = 3). (**c**) Clearing time comparison of A4P4B0.05-processed brain and A4P0B0-processed brain in PRE-CLARITY clearing (n = 3). (**d**) Intact brain Immunostaining time comparison of passive staining and CEx staining. Error bars in all histograms denote standard deviation.

**Figure 3:**
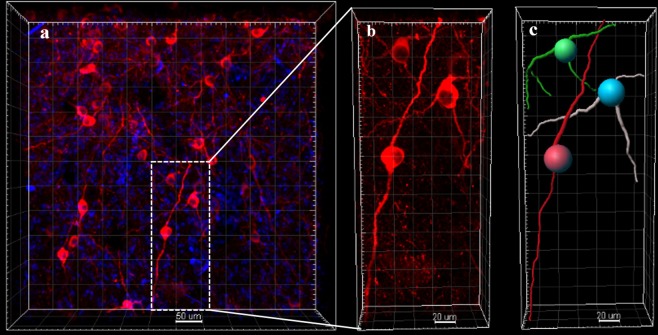
Images of electrophoresis cleared A4P0B0-processed brain. (**a**) The image was acquired by confocal microscopy (20x/0.75 dry objective). It shows dopaminergic neurons (stained with Anti-Tyrosine Hydroxylase antibody) at a part of the midbrain. (Red: anti-TH; blue: DAPI; Scale bar: 50 μm; square width: 50 μm). (**b**) The image shows neurons in the white boxed region of figure a. (Scale bar: 20 μm; square width: 20 μm). (**c**) The image shows dendrites in figure b. It was reconstructed with the filament auto-path toolkit in Imaris 9.0.1 software (Bitplane) (Scale bar: 20 μm; square width: 20 μm).

In electrophoresis clearing, the A4P4B0.05-processed brain showed that it is difficult to achieve transparency in our system, and the transmittance of the brain is only 30% even after 5 days of clearing (Fig. 1b,h). The cleared brain could not be imaged with a confocal microscope. Compared with our modified electrophoresis clearing using the A4P0B0-processed brain, the A4P4B0.05-processed brain was less transparent though it had been cleared for a much longer time (Fig. 1h; Fig. 2a). The transmittance of the A4P4B0.05-processed and A4P0B0-processed brain was 30% and 48%, respectively (Fig. 1h), and there was a significant difference between them (p < 0.005, n = 3). The image of the A4P0B0-processed brain also showed clear neuronal axons and dendrites (Fig. 3a–c). It demonstrated our modifications (including A4P0B0 embedding method and adding a-thioglycerol) could achieve faster clearing and higher transparency than the A4P4B0.05-processed brain when performing electrophoresis clearing.

Moreover, the clearing speed was different when using different PFA post-fixation times. The post-fixation time is usually determined empirically. Thus we explored the effect of different post-fixation times on clearing speed. As the most common post-fixation times are 12 hours and 24 hours, we tested these two fixation times and even shortened the time to 10 hours (Supplementary Fig. S2). The brain expanded and became more homogeneous with less opacity when using shorter time post-fixations. There was an obvious difference in expansion and degree of opacity even between the brains after 10 and 12 hours post-fixation. This showed that the clearing speed was highly related to PFA post-fixation time. When using a post-fixation time shorter than 10 hours (we tested 6 hours and 8 hours post-fixation for intact mouse brain), the brain was easily destroyed during electrophoresis or staining procedure.

In addition, the brain became yellow easily during electrophoresis because of the Maillard reaction. It was reported α-thioglycerol could address this problem in PACT^13^. We tested with and without α-thioglycerol in the clearing buffer when performing electrophoretic clearing of the A4P0B0-processed brain. After clearing for the same time, the brain with 1% α-thioglycerol added to the clearing buffer was white and transparent, while the brain without α-thioglycerol was yellow (Supplementary Fig. S3). Further, in passive clearing of the A4P0B0-processed brain, α-thioglycerol enhanced clearing speed. We performed A4P0B0-processed brain clearing with or without 5% α-thioglycerol. The clearing time was shortened to 14 days with the addition of 5% α-thioglycerol, while it required 20 days without α-thioglycerol. Therefore, α-thioglycerol worked well to prevent brain coloration during electrophoretic clearing and accelerate the clearing speed in passive clearing.

**Figure 4:**
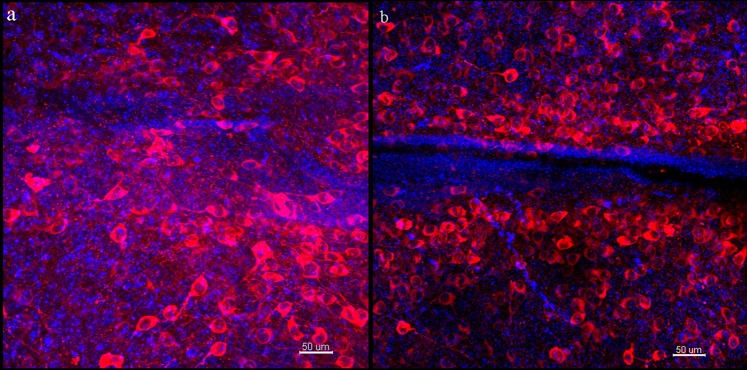
Images of passive and PRE-CLARITY cleared A4P4B0.05-processed brain. (**a**) The image was acquired by confocal microscopy (20x/0.75 dry objective). It is a projection of image stacks and shows stained dopaminergic neurons in the passive cleared A4P4B0.05-processed brain (Red: anti-TH; blue: DAPI; Scale bar: 50 μm; z stack: 516.62 μm). (**b**) The image was acquired by confocal microscopy (20x/0.75 dry objective). It is a projection of image stacks and shows stained dopaminergic neurons in the PRE-CLARITY cleared A4P4B0.05-processed brain (Red: anti-TH; blue: DAPI; Scale bar: 50 μm; z stack: 698.5 μm).

**Figure 5:**
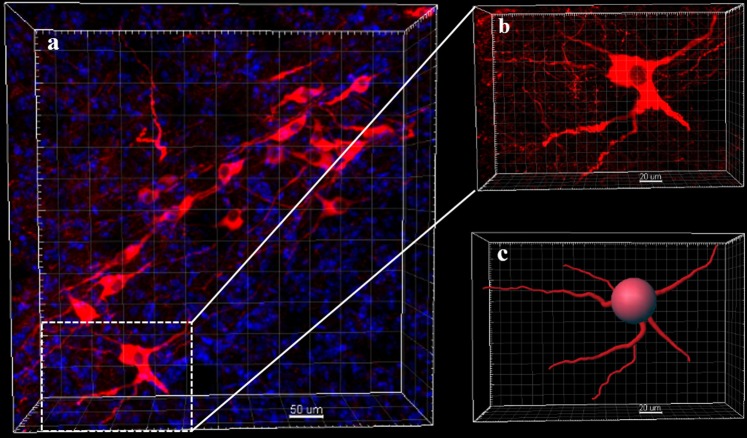
Images of passive cleared A4P0B0-processed brain. (**a**) The image was acquired by confocal microscopy (20x/0.75 dry objective). Dopaminergic neurons stained with Anti-Tyrosine Hydroxylase antibody. (Red: anti-TH; blue: DAPI; Scale bar: 50 μm; square width: 50 μm). (**b**) The image shows neurons in the white boxed region of figure a. (Scale bar: 20 μm; square width: 10 μm). (**c**) The image shows neuronal dendrites in figure b. It is reconstructed with the filament auto-path toolkit in Imaris 9.0.1 software (Bitplane) (Scale bar: 20 μm; square width: 10 μm).

As for passive clearing, both the A4P4B0.05-processed brain and A4P0B0-processed brain were transparent and were not colored (Fig. 1d,e). Both images of the brains showed a good resolution of dopaminergic neurons that were labeled (Figs 4a and 5a–c). However, the transmittance of the A4P4B0.05-processed and A4P0B0-processed brain was 51% and 55%, respectively (Fig. 1h), and there was a significant difference (p < 0.05, n = 3). It demonstrated that the A4P0B0-processed brain was easier to clear than the A4P4B0.05-processed brain in passive clearing. However, the A4P4B0.05-processed brain needed 1 month to achieve intact brain clearing while the modified A4P0B0-processed brain only needed 14 days in our study (Fig. 2b). Furthermore, the A4P4B0.05-processed brain was cleared at 50 °C, but the A4P0B0-processed brain was cleared at 37 °C. Therefore, our modified A4P0B0-processed passive brain clearing not only resulted in faster clearing speed but also achieved transparency at a mild temperature to avoid autofluorescence bleaching.

### PRE-CLARITY achieves higher transparency

**Figure 6:**
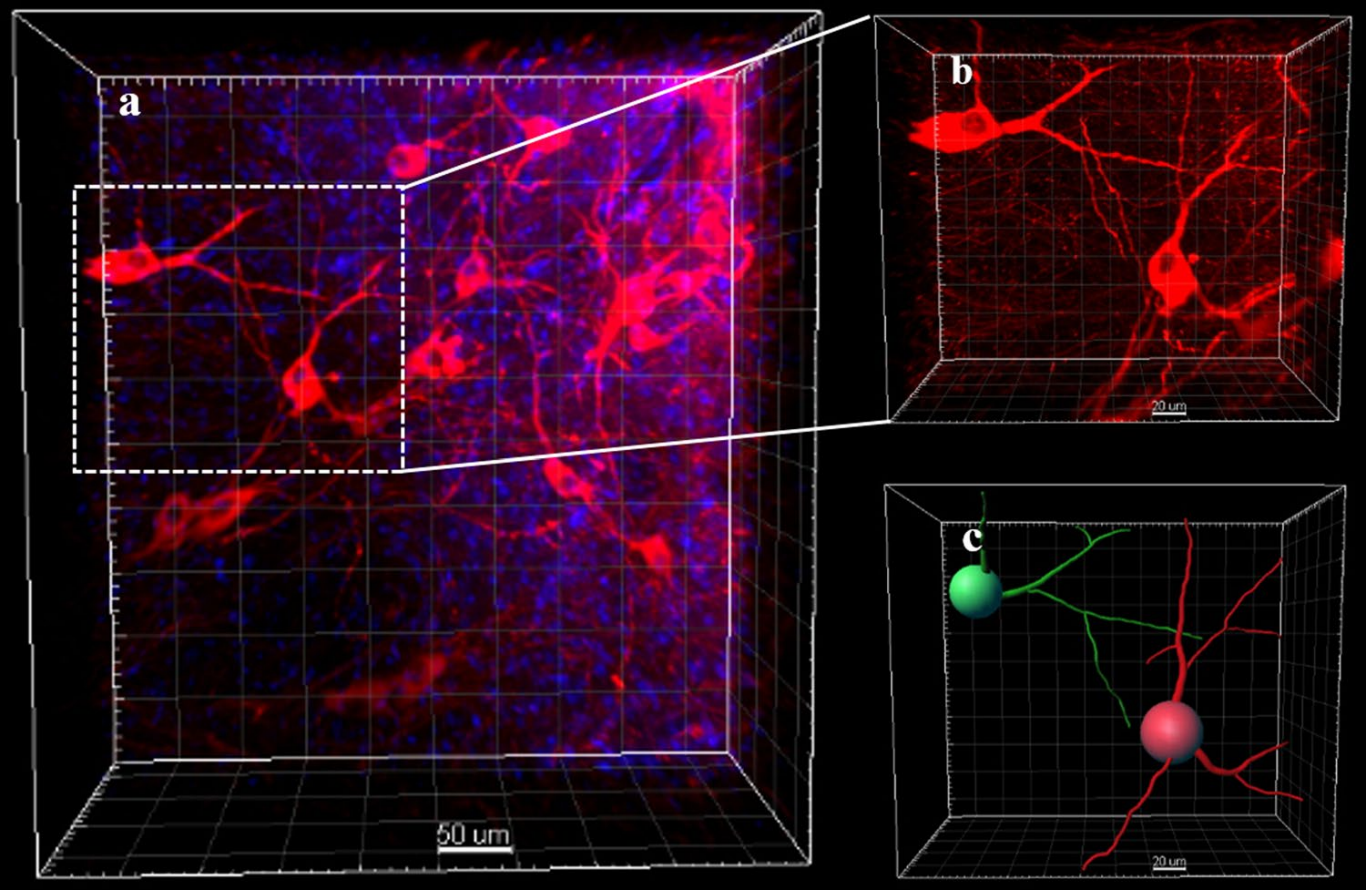
Images of PRE-CLARITY cleared A4P0B0-processed brain. (**a**) The image was acquired by confocal microscopy (20x/0.75 dry objective). It shows dopaminergic neurons stained with Anti-Tyrosine Hydroxylase antibody. (Red: anti-TH; blue: DAPI; Scale bar: 50 μm; square width: 50 μm). (**b**) The amplificatory 3D view of neurons in the white boxed region of figure a. (Scale bar: 20 μm; square width: 20 μm). (**c**) The image shows neuronal dendrites in figure b. It is reconstructed with the filament auto-path toolkit in Imaris 9.0.1 software (Bitplane) (Scale bar: 20 μm; square width: 20 μm).

In passive clearing, it is hard to achieve transparency in the central area of the brain. Generally, the brain quickly becomes transparent except for the central area, and extended times are needed to clear this area. First, we hypothesized that it was because the distance between this area and the clearing buffer is greater than other parts of the brain, and the clearing buffer did not easily reach the central part as the surrounding structure obstructed its penetration. So we set the passive clearing with a 2-mm brain A4P0B0-processed coronal slice of the central part. In the brain slice, this area was directly contacted by clearing buffer, and the distance between each brain part and the clearing buffer is the same. However, though the brain slice was almost cleared after 4 days of clearing, the central area in accordance with the less transparent area of intact brain was still opaque (Supplementary Fig. S4a). So it is not the distance that makes the central area hard to clear. Moreover, we found that the brain would expand and become homogeneous after a certain duration of electrophoresis while the brain processing passive clearing not (Supplementary Fig. S4b). Then we tried to perform a short time electrophoresis before passive clearing and interrogate whether the electrophoresis processing could promote the next passive clearing. Both the A4P4B0.05-processed brain and the A4P0B0-processed brain were subject to electrophoresis clearing for 1 day and then switched to passive clearing. After electrophoresis, the brains expanded and became homogeneous but were still opaque (Fig. 1f,g). After passive clearing, both the A4P4B0.05-processed brain and the A4P0B0-processed brain showed higher transparency than those processed with individual electrophoresis or passive clearing (Fig. 1f–h). For the A4P4B0.05-processed brain, it took 16 days (1 day electrophoresis and 15 days passive clearing) to achieve higher transparency (transmittance 61%), while it required 1 month for individual passive clearing with only 51% transmittance achieved (p < 0.005, n = 3) (Fig. 1h). The image of the PRE-CLARITY cleared A4P4B0.05-processed brain also showed a clear resolution of stained neurons (Fig. 4b). The transmittance of electrophoresis cleared A4P4B0.05-processed brain was only 30% (p < 0.001, n = 3) (Fig. 1h), and it could not be imaged with confocal microscopy. For the modified A4P0B0-processed brain, there was no obvious difference in transmittance between the PRE-CLARITY and passive cleared brain. The transmittance of PRE-CLARITY cleared brain is 57%, while it is 55% for the passive cleared brain (p > 0.05, n = 3). The images of the PRE-CLARITY cleared A4P0B0-processed brain showed dendritic arborization of dopaminergic neurons clearly (Fig. 6a–c). However, PRE-CLARITY shortened the clearing time to 6 days compared with 14 days in passive clearing (Fig. 2b,c). There was a significant difference in transmittance between the PRE-CLARITY (54%) and electrophoresis (45%) cleared brain (p < 0.005, n = 3) (Fig. 1h). In general, compared with individual electrophoresis or passive clearing, PRE-CLARITY enhanced the transparency for the A4P4B0.05-processed brain. As for the A4P0B0-processed brain, it showed significant improvements in transparency over electrophoresis clearing. Moreover, PRE-CLARITY shortened the clearing time to half compared with passive clearing while achieving higher transparency.

### Faster labeling with CEx staining

**Figure 7:**
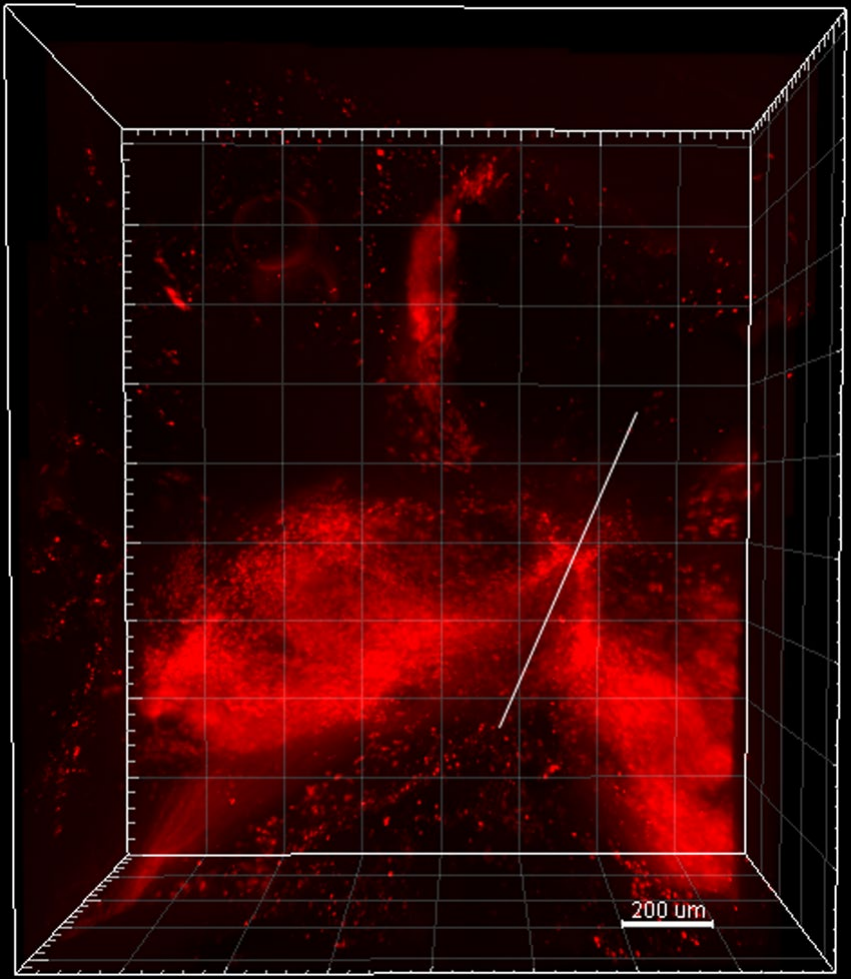
3D rendering of PRE-CLARITY cleared A4P0B0-processed brain. The image was acquired by lightsheet microscopy (5x/0.16 dry objective). The brain was stained with anti-TH antibody by CEx method. It shows the basal ganglia area has been labeled almost entirely. For some reasons the sample is not at the central position and the white line shows the midline of brain (Scale bar: 200 μm; square width: 200 μm).

In our study, we first found that 0.2 M boric acid solution expanded the cleared brain during washing. We considered that it could make the pores of hydrogel network increase in size, and antibodies could penetrate into the deep areas easily. To make it more suitable for antibody staining, we adjusted the pH to 7.2 and then tested the effects on brains cleared by the different ways (electrophoresis, passive and PRE-CLARITY clearing). All brains expanded after 0.2 M boric acid buffer (pH 7.2) processing for 12 hours at 37 °C (Supplementary Fig. S5). In order to prove the penetration promotion of 0.2 M boric acid buffer, we performed antibody penetration depth test. In our study, after 3 hours centrifugation, the penetration depth is 133 μm when using PBST as antibody diluting solution while it’s 180 μm when using boric acid buffer (Supplementary Fig. S6). Then, we performed centrifugation on the brains with or without boric acid buffer in the same conditions. With boric acid buffer, the intact brain staining time was 24 hours for each antibody staining, while it required 40 hours without boric acid buffer. Compared with passive staining, CEx staining is also much faster (Fig. 2d). We selected and reconstructed a part with neurons from each image of C-E stained brain (Figs 3b, 5b and 6b) and traced the neuron dendrites with Imaris software (Figs 3c, 5c and 6c). All of them showed clear dendrites in our study. Moreover, we imaged the A4P0B0-processed PRE-CLARITY cleared brain with lightsheet microscopy. It shows the deep brain (Basal ganglia) has been labeled almost entirely (Fig. 7). In general, CEx staining achieved faster staining than original passive staining or individual centrifugation staining.

### Choosing of RI matching solution

When choosing the RI matching solution, many parameters such as viscosity, dissolubility and the ability of anti-swelling should be considered. For large-scale samples processed with CLARITY, the ability of anti-swelling is extremely important because of the limitation of microscope working distance and imaging depth. In our study, We tested the anti-swelling ability of 88% histodenz^9^ (RIMS) and 63% 2,2′-thiodiethanol^15^ (TDE). Compared to the brain after 63% TDE and RIMS processing, 63% TDE showed better anti-swelling ability than RIMS (Supplementary Fig. S7). Therefore, we adopted 63% TDE as the RI matching solution.

## Discussion

Tissue optical clearing technique have developed quickly these years, and many new clearing techniques emerged gradually. Different clearing techniques have different indications for various specimens^16,17^. CLARITY creates hydrogel-tissue hybridization and fixes natural molecules at their position while extracting lipids to achieve tissue transparency. It has high tissue compatibility and permits several rounds of immunostaining^2,6^. However, the complex electrophoresis system makes it hard to manipulate, and the long processing time required for immunostaining also impedes its wide application in more fields.

In our study, we created a new electrophoresis system named NCES, which does not require circulation and is easier to make and manipulate during electrophoresis. By removing the circulation system, many additional devices are also unnecessary such as the peristaltic pump, filter, and refrigerated circulator. It not only makes electrophoresis tissue clearing easier to handle but also saves money. The easy-made mobile electrophoresis device in NCES could be modified to a different shape to suit various samples. Additionally, even the electrophoresis device could be separated into several parts, and the electrodes can be combined with different size pedestals as needed so that a pair of electrodes could be applied to many researches. Compared with other CLARITY modifications to electrophoresis system, such as changing electrophoresis chamber^11,18^, our study provided a more effective way to simplify it and it is more flexible to suit different size of sample. However, in our NCES system we adopted platinum wire to make electrodes, while it’s reported platinum plate could generate a more dense current and accelerate the clearing speed^11^. It could be tested in the next work. And there is another negative result in our study that the A4P4B0.05-processed brain didn’t be cleared well with NCES. In the article reported by *Tomer et al*.^4^, they recommended running the ETC process at 25 V and 37 °C for ~3–5 d when performing adult mouse brain sample clearing. But we couldn’t achieve intact brain clearing in 5 days (it seems far from clearing). Comparing the original electrophoresis system and the NCES, the main difference is the flow of clearing buffer. So we considered that flow-assisted dissociation of lipid molecules also plays an important role in tissue clearing. And as reported in another article^6^, they accomplished intact adult mouse brain clearing in 12–16 days at constant current 280 mA with varying voltage 22–26 V. So the electrophoresis clearing time might be extended. But in different articles, authors adopted different PFA or hydrogel monomers post fixation time which would affect the hydrogel structure. And the age of mice also various which results in different physical size and myelinization of brain^2,4,13^. So these aspects also do affect to the clearing speed and should be considered when performing brain clearing.

It has been reported that combining different temperatures in tissue clearing with CLARITY achieves higher transparency and faster clearing speeds than individual temperature processing^19^. In our study, we developed a combination of electrophoresis and passive clearing, which we named PRE-CLARITY. It also showed that PRE-CLARITY promote clearing speed and improved transparency. Electrophoresis provides additional electric fields which give stronger forces to eliminate lipids, and it is gently affected by the tissue structure compared with passive clearing^20–22^. The samples after electrophoresis clearing would expand more and become more homogeneous compared with those after passive clearing. But the strong forces also reduced the tissue rigidity, and the sample may not afford a long passive clearing after that, which would also restrict obtaining higher tissue transparency. It is also why the PRE-CLARITY cleared A4P4B0.05-processed brain showed higher transmittance than the A4P0B0-processed brain. Thus, there is a trade-off between electrophoresis time and tissue transparency (or tissue rigidity). In our work, we compared A4P4B0.05 and A4P0B0 processed brain clearing with electrophoresis. And more different concentration of acrylamide and PFA might be tested in the next work as described in the work of *Bastrup et al*.^18^. But in their work, they used A4P4, A4P0, A0P4 and A2P2 as perfusion solution, which is different from our work. Moreover, we only tested 1-day electrophoresis combined with passive tissue clearing, but different combinations of electrophoresis times and passive clearing times should be tested. And the natural properties of specimens should also be considered. Our study provides some new experiences for the combination of different clearing methods.

As for immunostaining, a major advantage of CLARITY is that it permits multiple rounds of immunohistochemistry labeling after clearing compared with other tissue clearing methods^2^. However, the long processing time impedes its wide application. Lee *et al*.^11^ had reported that applying centrifugation could accelerate immunostaining speed, but this only helped with dense tissues (their group tested kidney, testis, lung, and liver). In our study, we raised the rotation speed to 1200 r/min and extended the centrifugation time to achieve intact mouse brain labeling. Additionally, we also made the brain expand with boric acid buffer during washing and labeling, which resulted in better antibody penetration. CEx staining can achieve each antibody staining of intact mouse brain in one day, while one week is required with passive staining in the original CLARITY protocol^4^. It greatly shortens immunostaining time and could be applied in more fields to achieve large-scale specimen labeling. But we should pay attention to the antibody concentration when performing immunostaining. Higher antibody concentration is important especially when performing large tissue staining. We also used higher antibody concentration than that in ACT-PRESTO as we stained intact brain rather than tissue slices.

We also made some modifications to the PFA post-fixation time and clearing buffer. The post-fixation time is related to the rigidity of the sample, which affects the clearing speed directly. However, adjusting tissue rigidity should be related to different clearing and labeling methods. As the electrophoresis clearing time is much shorter than that of the passive clearing, we shortened the fixation time in the electrophoresis clearing brain but elongated it in passive clearing brain. However, we did not assess tissue deformation at a microscopic level or protein loss during electrophoresis. As reported there is also a trade-off between transparency and protein retention^23^, so it is better to modify the parameters related to hydrogel embedding while considering tissue deformation and protein loss. Moreover, we added α-thioglycerol into the clearing buffer to prevent brain yellowing and accelerate the clearing speed. This method has been reported before^13^ and was named mPACT. We adopted a higher concentration of α-thioglycerol and applied it to electrophoresis clearing while it was used in passive clearing with mPACT. It is also a combination of mPACT and electrophoresis clearing. And because α-thioglycerol is toxic and our electrophoresis clearing system is open, so we adopt lower concentration of α-thioglycerol in electrophoresis (1%) while it’s higher in passive clearing (5%). Moreover, it’s reported that the adding of α-thioglycerol could expedite the clearing speed in passive clearing^13^. And it’s also recommended to use in electrophoresis clearing^23^. In our study, we found the effect to clearing speed is also related to the concentration of α-thioglycerol. So we raise the concentration to several folds than that in mPACT. However, we did not investigate the effect of different concentrations of α-thioglycerol systematically, which might be accomplished in the next studies.

In terms of the RI matching solutions, a lot of solutions have been reported^2,9,11,15,24^. But authors rarely compared the anti-swelling capacity of them and only focused on the RI. However, especially for large tissues, the anti-swelling capacity is very important because of the work distance limitation of microscope. In our study, we compared RIMS (88% histodenz) and TDE (63% 2,2′-thiodiethanol). And both of them showed expansion after incubation, compared with tissue before clearing. It’s also showed expansion in the original articles^15,24^. But TDE showed better anti-swelling capacity than RIMS in our work. So we recommended to use TDE when performing large tissue imaging.

In general, our study provided an easy electrophoresis system and modified CLARITY protocol to achieve faster clearing and labeling as well as a higher degree of transparency. Moreover, our investigation into the combination of different clearing methods provides a new view of tissue optical clearing techniques. More clearing techniques could be tested and combined to solve practical problems.

## Methods

### Brain fixation and hydrogel embedding

All aspects of animal husbandry and experimentation were performed in accordance with guidelines from the Third Military Medical University and have been approved by members of the Third Military Medical University Institutional Animal Care and Use Committee.

Male C57BL/6 mice were purchased from the Third Military Medical University Animal Center. One-month-old mice were used for brain clearing.

#### A4P0B0-processed brain

Mice were deeply anesthetized and transcardially perfused with 20 ml PBS followed by 20 ml 4% PFA. Brains were excised and then post-fixed in 4% PFA at 4 °C. Brains for electrophoretic clearing were post-fixed for 10 hours, while those for passive clearing and PRE-CLARITY were post-fixed for 28 hours. Fixed brains were incubated in A4P0B0 hydrogel monomer solution (4% acrylamide (J&K scientific, 166974) in PBS) supplemented with 0.25% VA-044 initiator (J&K scientific, 503236) for 24 hours at 4 °C. A4P0B0-infused samples were degassed for 10 minutes and polymerized for 6 hours at 37 °C. Samples were washed several times in PBS before electrophoresis clearing.

#### A4P4B0.05-processed brain

The brains were processed as reported^4^. Briefly, mice were transcardially perfused with 20 ml A4P4B0.05 solution (4% acrylamide, 0.05% bis-acrylamide (AMERISCO, 0319B595), 4% PFA and 0.25% VA-044 initiator in PBS) and then incubated in A4P4B0.05 solution for 24 hours. After that, the brain was polymerized and extracted from solidified hydrogel. The brain was washed several times in PBS before electrophoresis clearing.

### Design of NCES

As showed in Fig. 1, we separated the electrodes from the electrophoresis chamber in the original CLARITY method and designed a mobile electrophoresis device. It does not need circulation as the clearing buffer is reserved in the beaker and it is put in a refrigerated water bath. After electrophoresis is started, bubbles formed continually, but they would stay at the top of the water level and then dissolve gradually. Bubbles would not escape from the beaker so long as the clearing buffer does not fill more than half of the beaker. Because there is no filter to eliminate byproducts, another barrier is needed to avoid byproducts such as some black micelle deposits on samples. We used qualitative filter paper to wrap the brain, and it worked well to prevent byproduct deposition.

### Brain clearing

#### Electrophoresis brain clearing

First, A4P4B0.05-processed brains were subject to electrophoresis clearing with NCES at 25 V, 37 °C as reported^4^. The clearing buffer was 4% SDS (Aladdin, S108346) in distilled water with 0.2 M boric acid buffer (pH adjusted to 8.5). When performing A4P0B0-processed brain, 1% α-thioglycerol (Aladdin, T109440) was added to 4% SDS and 0.2 M boric acid clearing buffer. During electrophoresis, the clearing buffer was refreshed after two days. Although the buffer pH did not drop below 7.5, the buffer color turned yellow, and it would color the brain. So it is better to refresh the clearing buffer according to the buffer color rather than the pH. Electrophoresis of A4P4B0.05-processed brain was stopped after 5 days, as it could no longer improve transparency (Fig. 1b), while it was stopped after 60 hours for A4P0B0-processed brain (Fig. 1c).

#### Passive brain clearing

The A4P4B0.05-processed brain was cleared as reported^4^. The brain was put into a 50 ml conical tube with 4% SDS and 0.2 M boric acid clearing buffer and gently shook at 50 °C. The clearing buffer was refreshed when the pH dropped below 7.5. After 1 month, the brain became transparent (Fig. 1d). As for the A4P0B0-processed brain, 5% α-thioglycerol was added into the clearing buffer, and the brain was cleared at 37 °C. After 14 days, the brain became transparent (Fig. 1e).

#### PRE-CLARITY Brain clearing

First, the A4P4B0.05-processed brain was cleared by NCES for 1 day with 4% SDS clearing buffer containing 1% α-thioglycerol. Then, passive clearing was conducted in 4% SDS and 5% α-thioglycerol clearing buffer at 50 °C, with gentle shaking. The brain was totally transparent after 15 days (Fig. 1f). For the A4P0B0-processed brain, we also performed electrophoresis with NCES for 1 day and then started passive clearing with the same clearing buffer. After 5 days, the brain became transparent (Fig. 1g).

### Transmittance measurement of cleared brains

The cleared brains were subject to transmittance measurements with a microplate spectrophotometer (Tecan Infinite® 200Pro). The specimen was put in the microplate, and the same clearing buffer used in the clearing process was added to submerge it. The clearing buffer was added to fill the well plates to equal heights. Firstly we measured its absorbance with an incident laser of 400–900 nm wavelength and calculated the transmittance by the Lambert-Beer law (${\rm{T}}\,=\,{\rm{log}}1/A$; A = absorbance, T = transmittance). We found that the transmittance of brain increased with the laser wavelength increasing (Supplementary Fig. S8). And because the emission wavelength of our secondary antibody is 580 nm. So we chose 580 nm wavelength incident laser to calculate the brain transparency.

### Immunostaining of cleared brains

#### Passive immunostaining

We applied passive immunostaining to A4P4B0.05-processed brains (including electrophoresis, passive and PRE-CLARITY cleared brain) as reported^4^. Cleared brains were washed with PBST at 37 °C with gentle shaking for 2 days (refresh PBST every 4–6 hours). Then, the specimen was incubated with anti-tyrosine hydroxylase antibody solution (1:30, Abcam, ab112) including 5% goat serum, 0.1% Triton X-100 and 0.05% proclin 300 in PBS at 37 °C with gentle shaking. The whole mouse brain was incubated in 3 ml solution and 50 µl anti-TH antibody was added at the first day, followed by 30 µl at the third day and 20 µl at the fifth day to reach a 1:30 final concentration. After 1 week, the primary antibody was washed with PBST at 37 °C for 3 days (refresh PBST every 4–6 hours). Then, we performed Alexa Fluor 555 antibody (1:30, Bioss, bs-0295G-AF555) staining and washed it with the same procedure as primary antibody washing. Nuclear labeling dye DAPI (1 µg/ml, Beyotime, C1006) was added on the last day during secondary antibody staining.

#### CEx staining

We first applied centrifugation to the A4P0B0-processed electrophoresis-cleared brain. The brain was washed by PBST with centrifugation. The brain was transferred to a 50 ml conical tube with PBST and centrifugation was performed (1200r/min) for 5 hours (refreshed PBST each hour). After washing, the brain was incubated with anti-TH antibody (1:30, Abcam, ab112) solution including 5% goat serum, 0.1% Triton X-100 and 0.05% proclin 300 in PBS. The antibody was added in one step at the beginning to reach a 1:30 concentration, rather than in separate steps. Then, we centrifuged the brain for 40 hours. After that, it was washed with centrifugation (1200 r/min) for 5 hours (refreshed PBST each hour). Then, it was processed with Alexa Fluor 555 antibody staining (1:30) and washed with the same procedure as primary antibody staining. DAPI (1 µg/ml) was added in the last 12 hours during secondary antibody staining.

In addition, we found that boric acid solution expanded the cleared brain (Supplementary Fig. S4). So we considered that it might increase the hydrogel network pore size potentially, and then antibodies penetrate into deep tissue easier. Therefore, we used 0.2 M boric acid buffer (sodium hydroxide adjusts pH to 7.2) to perform brain washing and staining, a protocol that we named CEx staining. PBST in the washing procedure and PBS in the staining procedure were substituted with 0.2 M boric acid buffer (pH 7.2). To verify the penetration promotion of boric acid buffer, we performed an antibody penetration depth test. Two 1 mm A4P0B0-processed brain slices were acquired from two brains in order to get slices at the same position. Then brain slices were cleared by passive clearing protocol as descripted in the 3.2 part above. After clearing, two brain slices were washed by PBST or 0.2 M boric acid buffer (pH 7.2) individually. Then we performed Anti-NeuN/AF488 Conjugated antibody (1:40, abcam, ab190195) and DAPI (1: 1000) staining using centrifugation. Two brain slices were incubated in antibody solutions which were diluted with PBST or 0.2 M boric acid buffer individually. After 3 hours centrifugation, brain slices were washed by PBST or 0.2 M boric acid buffer individually.

Next, we applied centrifugation (1200 r/min) to A4P0B0-processed electrophoresis, passive and PRE-CLARITY cleared brains. The cleared brains were subjected to 24 hours anti-TH antibody (1:30) and Alexa Fluor 555 antibody staining (1:30) individually. DAPI (1 µg/ml) was added in the last 12 hours during secondary antibody staining.

### Refractive index (RI) matching and imaging

After immunostaining, the cleared brain was incubated with 63% 2,2′-thiodiethanol(TDE) (Sigma, 166782) in PBS for 24 hours at room temperature. Then, the brain was imaged with confocal microscopy (Leica SP8) or lightsheet microscopy (ZEISS, Z1) and processed with Imaris 9.0.1 software (Bitplane).

### Statistical analysis

We performed two-tailed t-test analysis to compare the degree of transparency, and the alpha level for all experiments was 0.05.

## Supplementary information

supplementary information

## Data Availability

Data used in this study are available upon reasonable request.
