# Supplementary material for: Modified CLARITY Achieving Faster and Better Intact Mouse Brain Clearing and Immunostaining
Source: Sci Rep. 2019 Jul 22;9:10571. doi: 10.1038/s41598-019-46814-4 (PMC6646319; doi:10.1038/s41598-019-46814-4)
Supplement: Supplementary file 1 — supplementary information [file 41598_2019_46814_MOESM1_ESM.pdf]

Modified CLARITY Achieving Faster and Better Intact Mouse Brain Clearing and Immunostaining

Hao Du[1*], Peihong Hou[1*], Liting Wang[2], Zhongke Wang[3], Qiyu Li[1#]

1： Department of Anatomy, Third Military Medical University, Chongqing, China, 400038

2： Biomedical Analysis Center, Third Military Medical University, Chongqing, China, 400038

3： Department of Neurosurgery, Xinqiao Hospital, Third Military Medical University, Chongqing, China, 400037

*: These authors contributed equally to this work.

#: corresponding author: Professor Qiyu Li, Department of Anatomy, Third Military Medical University, NO.30 of Gaotanyan Central Street, Shapingba, Chongqing 400038, P.R. China

Email: liqiyu_99@163.com

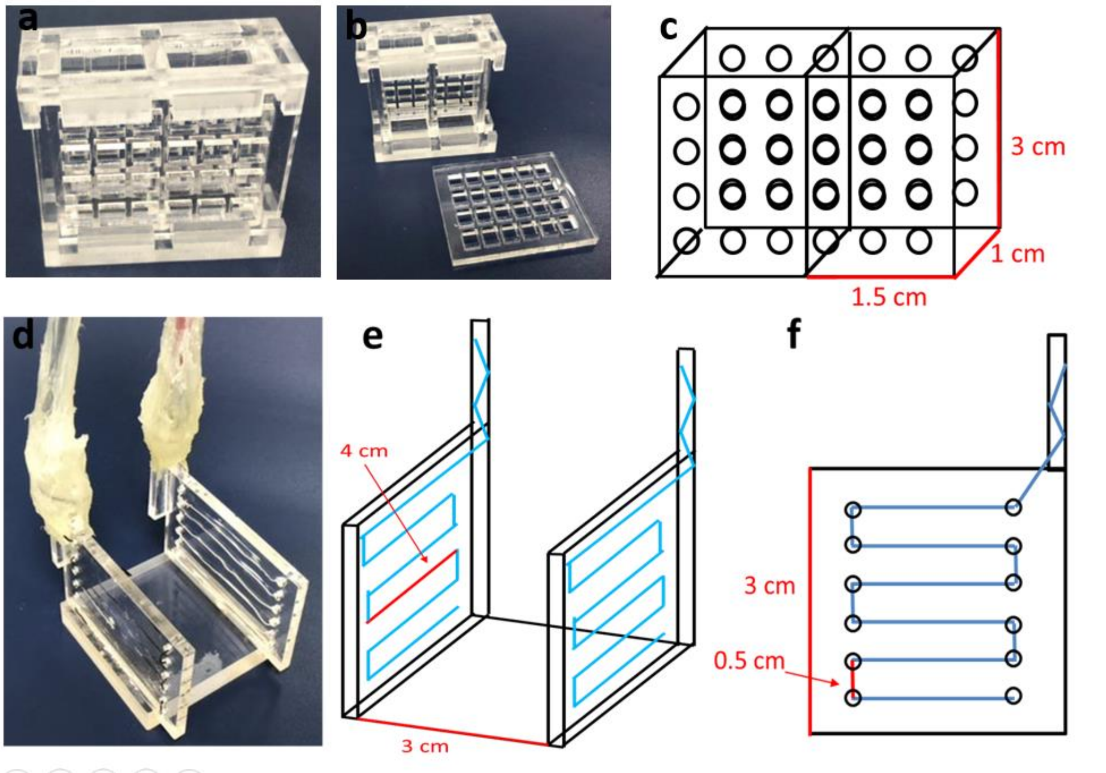

Supplementary figure S1. The real projects and dimensions of devices in NCES. (a-b) the real project of sample chamber. It shows the chamber when it's closed or opened. (c) It's the schematic picture of sample chamber. The red marks shows the inner length, width and height of sample chamber for one sample are 1.5 cm, 1 cm and 3 cm individually. The dimensions of the left part is the same to the right one. (d) It shows the real project of electrophoresis device. (e) It is the schematic picture of electrophoresis device. The blue line shows electrodes. The red marks shows the inner distance between two electrodes plane is 3 cm and the electrode length in each horizontal line is 4 cm. (f) It is the schematic picture of each electrode attached plate. The blue line shows electrodes and it is set continually from the bottle to top of the plate. The black circles denote small holes in the plate. The platinum wire passes through these holes to keep the wire

attached to plate stably. The red marks shows the height of the plate and the vertical part of electrodes are 3 cm and 0.5 cm individually.

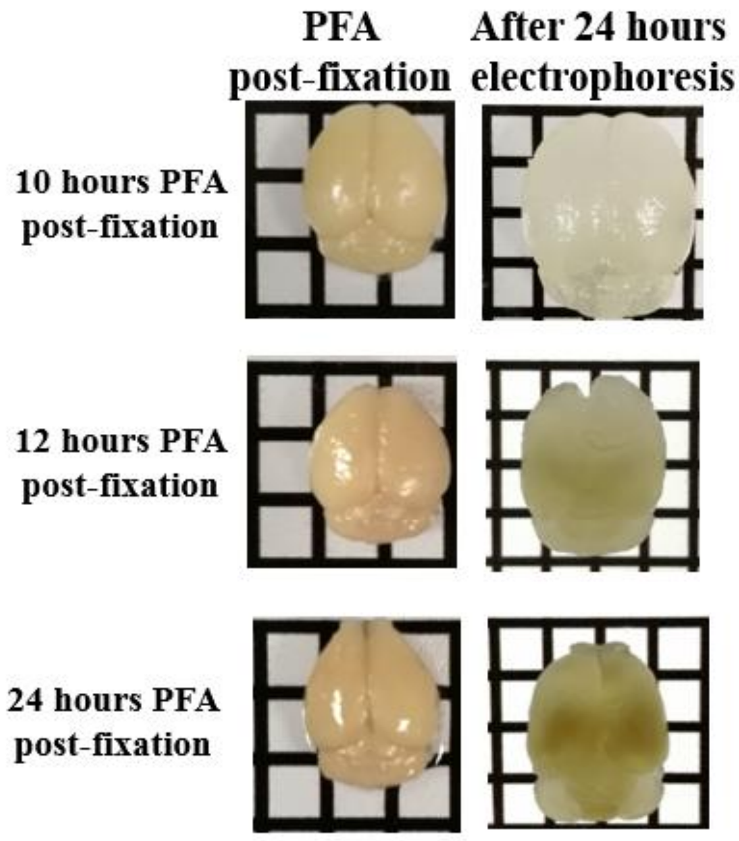

Supplementary figure S2. The comparison of electrophoresis processed brain after different times of PFA post-fixation. The top row shows 10 hour the PFA post-fixation brain before (left) and after (right) 24 hours of electrophoresis clearing. The middle row shows 12 hour PFA post-fixation brain before (left) and after (right) 24 hours of electrophoresis clearing. The bottom row shows 24 hours PFA post-fixation brain before (left) and after (right) 24 hours of electrophoresis clearing. All brains were embedded with A4P0 solution for 24 hours.

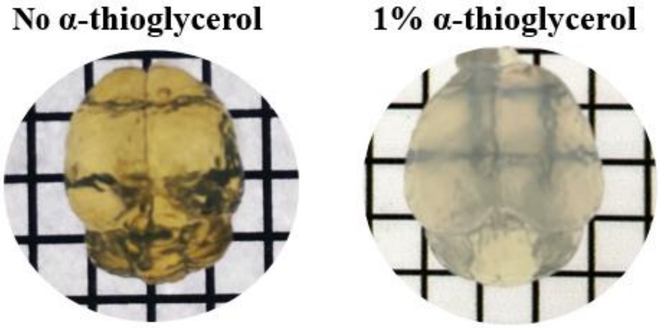

Supplementary figure S3. Comparison of electrophoresis cleared brain with (right) and without (left) adding 1% α-thioglycerol in clearing buffer. These two brains were embedded with A4P0 (10 hours PFA post fixation) and cleared by NCES at 37 ℃/25 V for 60 hours.

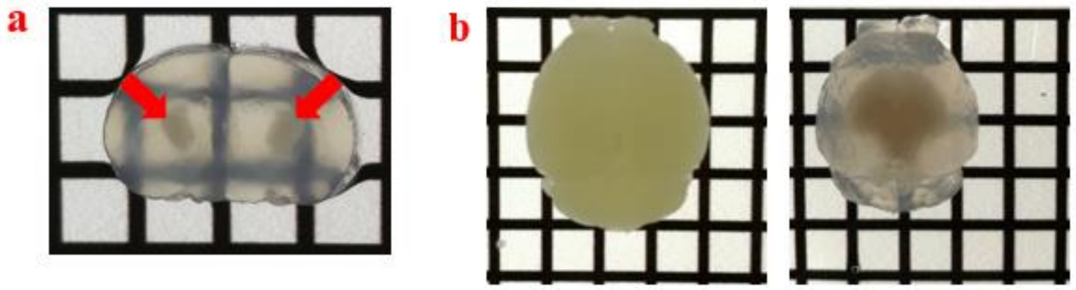

Supplementary figure S4. Electrophoresis expands the brain and it becomes more homogeneous than that in passive clearing. (a) It shows 28 hour PFA post-fixed A4P0-processed brain slice after 4 days passive clearing with 4% SDS containing 5% α-thioglycerol clearing buffer. The red arrows designate the opaque area after 4 days of passive clearing. (b) The left picture shows the A4P0-processed brain after 1 day of electrophoresis clearing with 4% SDS containing 1% α-thioglycerol clearing buffer. The right picture shows A4P0-processed brain after 6 days passive clearing with 4% SDS containing 5% α-thioglycerol clearing buffer.

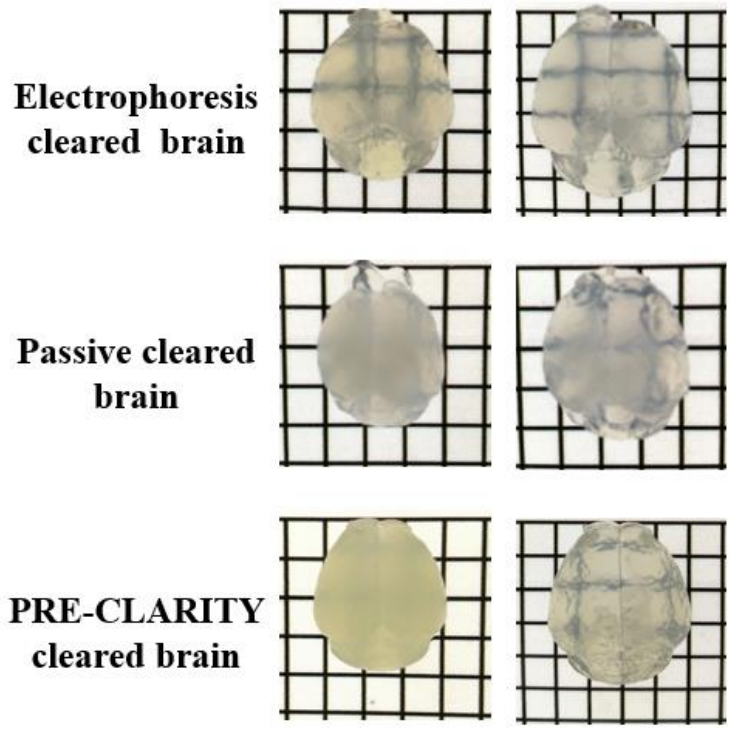

Supplementary figure S5. Comparison of different methods of cleared brains before and after boric acid buffer (pH 7.2) processing. The top, middle and bottom row shows the electrophoresis cleared brain, passive cleared brain, and PRE-CLARITY cleared brain individually. All brains were A4P0-processed. The left side shows brains before boric acid buffer processing and the right side shows brains after processing. (Note: the pictures of passive and PRE-CLARITY cleared brains before processing were not washed after clearing and had been stored in PBST at 4 ˚C for some time. So the residual SDS made them not transparent.)

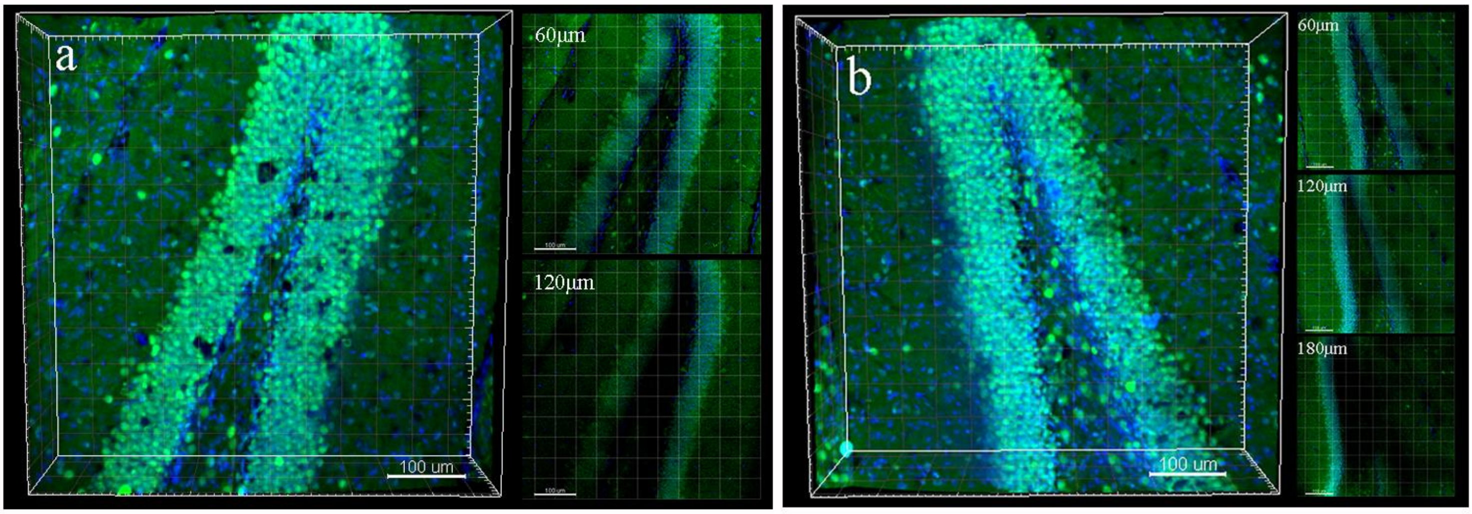

Supplementary figure S6. The penetration depth comparison of cleared brain slices staining with different antibody dilute solution. The image was acquired by confocal microscopy (20x/0.75 dry objective). The brain slices are stained with Anti-NeuN antibody (1:40, green) and DAPI (1:1000, blue) using centrifugation and images show the DG of hippocampus. (a) The slice is stained by using PBST to dilute antibody. The left image shows the 3D view of stained area and the images in the right show the images at 60 µm and 120 µm (scale bar: 100 µm; square width: 50 µm; Z stack: 133 µm). (b) The slice is stained by using boric acid buffer (pH 7.2) to dilute antibody. The left image shows the 3D view of stained area and the images in the right show the images at 60 µm, 120 µm and 180 µm (scale bar: 100 µm; square width: 50µm; Z stack: 180 µm).

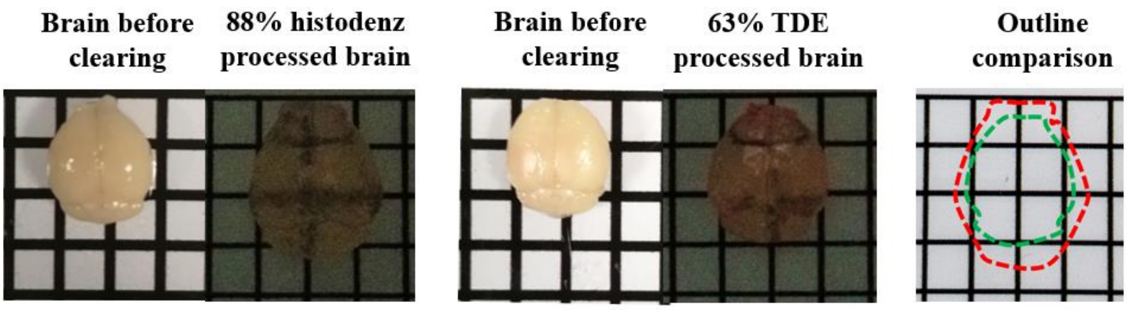

Supplementary figure S7. Comparison of brain after 88% histodenz or 63% TDE processing. The left pair of pictures shows the brain before clearing and after 88% histodenz processing. The middle pair of pictures shows the brain before clearing and after 63% TDE processing. The right picture shows the outline of brain after histodenz (red line) or TDE (green line) processing.

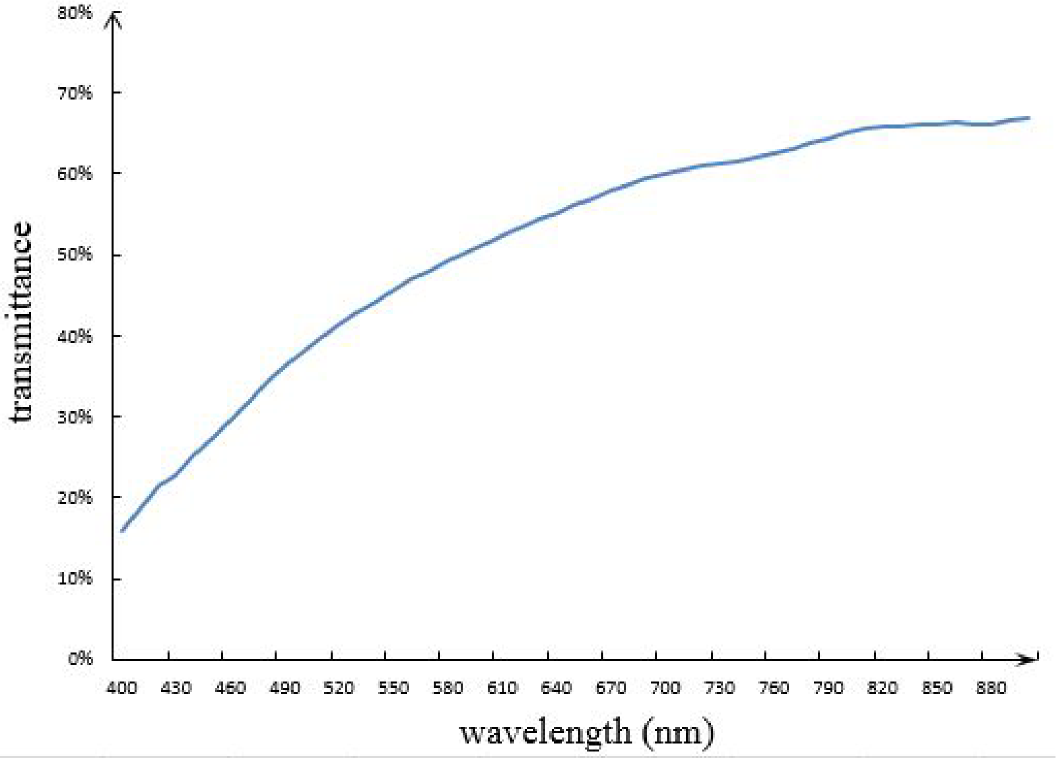

Supplementary figure S8. The transmittance of cleared brain in different incident laser wavelength. It shows the transmittance of A4P0-processed brain after electrophoresis clearing in 400 to 900 nm incident laser wavelength. The transmittance increased as the increasing of incident laser wavelength.
